# Knowledge mapping of exosomes in preeclampsia: a bibliometric analysis (2008-2023)

**DOI:** 10.3389/fendo.2025.1546554

**Published:** 2025-03-04

**Authors:** Liang Yin, Yuchao Zhang, Guanfeng Fu, Haiqin Huang, Hang Su, Yipeng Zhang, Qichun Chen, Qinghua Li, Weiwei Yang

**Affiliations:** ^1^ Department of Emergency, Affiliated Hospital of Shandong Second Medical University, Weifang, China; ^2^ School of Life Science and Technology, Shandong Second Medical University, Weifang, China; ^3^ School of Public Health, Shandong Second Medical University, Weifang, China

**Keywords:** preeclampsia, exosome, bibliometric, knowledge-map, CiteSpace, bibliometrix, VOSviewer

## Abstract

**Background:**

Exosome research in preeclampsia is gaining increasingly popular, however thorough and unbiased summaries of the field’s present understanding are hard to come by. Therefore, this study aims to conduct a bibliometric analysis of the publication “Exosomes in Preeclampsia” in order to visually analyze the state of the field and identify emerging trends.

**Methods:**

From 2008 to 2023, the Web of Science database was searched for publications related to exosomes in preeclampsia. Three software packages—VOSviewer, CiteSpace, and the R program “bibliometrix”—were used to conduct bibliometric analysis.

**Results:**

Analysis of 257 publications produced by 1454 scholars from 48 countries/regions and 435 institutions, published in 135 academic journals. The quantity of studies concerning exosomes in preeclampsia is steadily increasing. China and the United States lead in publications, with Oxford being the most active university. Placent has written the most relevant study and has received the highest number of citations. Carlos Salomon has the most number of published articles and is the most referenced author. The 10 most frequently mentioned sources were used as a knowledge basis. The predominant terms examined include extracellular vesicle, expression, pregnancy, microparticle, and microRNA. Utilizing fundamental research on exosomes in preeclampsia for clinical diagnosis and therapy is a current popular research focus and direction. Utilizing fundamental research on exosomes in preeclampsia for clinical diagnosis and treatment is currently a popular research focus and direction.

**Conclusion:**

This study offers a comprehensive overview of trends and advancements in the research of exosomes in preeclampsia through bibliometrics. This material highlights the current research frontiers and trending directions, serving as a valuable reference for researchers in the subject.

## Highlights

- The bibliometric analysis methods are comprehensive and accurate.
- The research hotspots and frontiers are precisely grasped.
- The research trend of exosomes in preeclampsia is clearly presented.
- The analysis of the basic knowledge is in-depth.

## 1 Introduction

Preeclampsia is a pregnancy complication that typically emerges after 20 weeks of gestation. This condition is mostly identified by elevated blood pressure and proteinuria, and may result in consequences for both the mother and fetus, including renal failure, cerebral hemorrhage, liver failure, and restricted fetal development (1). It is related with 10% of maternal impairments and fatalities worldwide. Preeclampsia primarily involves aberrant placenta development, with clinical signs often emerging during the second trimester of pregnancy, posing challenges for early management (2). Preeclampsia can only be effectively treated by terminating the pregnancy early (3). Preeclampsia is becoming a significant worldwide danger to maternal health.

Exosomes are small extracellular vesicles, typically measuring between 30-150 nm in diameter, found in the blood and other bodily fluids. Their composition includes lipids, proteins, RNA, DNA, and other biological components (4). Exosomes have shown potential as indicators and therapeutic targets for various applications in treating disorders including preeclampsia (5).

Bibliometrics is a technique for analyzing literature that examines the production and impact of publications in a certain topic using quantitative and qualitative approaches. Detailed information on authors, keywords, journals, countries, institutions, references, etc. in the specific study field may be acquired throughout the analysis process. Bibliometrics aims to identify the main contributors in a certain area of study, comprehend the present research status, and analyze research patterns and potential developments in the subject (6).When paired with visual analytics, bibliometrics is a powerful tool for academics to recognize patterns in knowledge and scholarly records, and to merge information (7). This research conducts a bibliometric analysis of exosomes in preeclampsia literature retrieved from the Web of Science Core Collection (WoSCC) database spanning from 2008 to 2023. This study aims to provide scholars guidance for future research.

## 2 Materials and methods

### 2.1 Data collection

**Figure 1:**
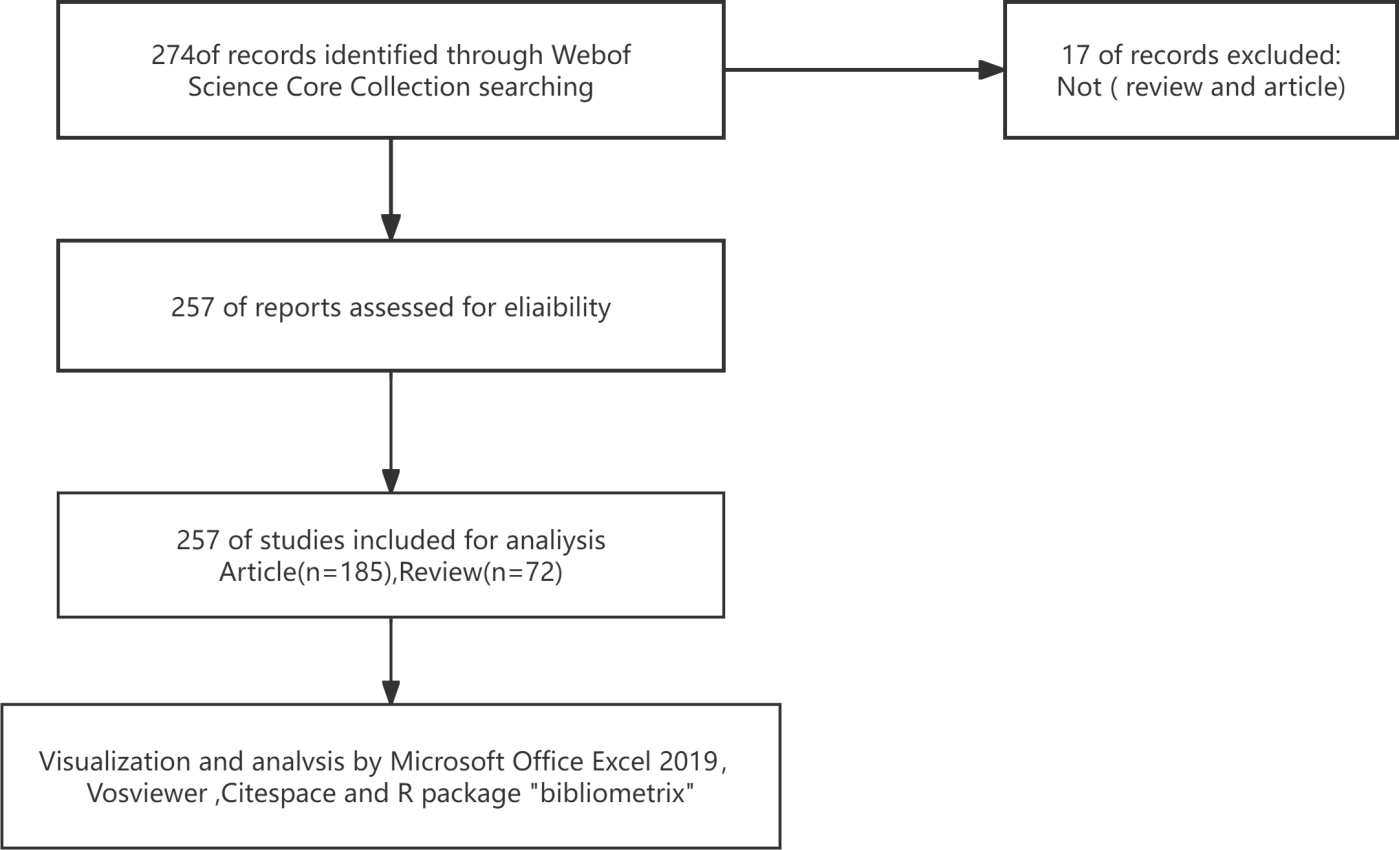
Flowchart outlining the data gathering process for exosomal research in preeclampsia.

We conducted a literature search on April 15, 2024, in the WoSCC database. The search strategy used terms related to exosomes and preeclampsia, specified the article type as articles and reviews, and set the time span from January 2008 to December 2023. The study analyzed publicly available datasets without requiring an ethical statement. A total of 257 items meeting the search criteria were identified and analyzed (**Figure 1**).

### 2.2 Data analysis

Analysis and visualization of bibliometric data were conducted using VOSviewer (v1.6.19), CiteSpace (v6.1.R6), Excel (v2019), and the R tool “bibliometrix” (v4.2.2).

VOSviewer is a bibliometric analysis tool capable of visualizing and analyzing network data to intuitively present knowledge structures and research trends. Compared to other similar tools, it excels in creating and visualizing knowledge maps commonly used for collaboration networks, co-citation networks, and co-occurrence networks (8). This advantage allows for a more intuitive and comprehensive understanding of complex relationships such as those between countries and institutions, authors and co-cited authors, journals and co-cited journals, and keyword co-occurrences, significantly enhancing research efficiency and accuracy. This study fully leverages the strengths of this tool to explore various aspects of the target literature.

CiteSpace is a bibliometric and scientific visualization analysis tool applied in research fields. Compared to other tools, through in-depth mining of knowledge units such as authors, institutions, countries/regions, keywords, and journals, it helps identify cooperative relationships, internal structures, key points, potential trends, and dynamic changes within scientific fields (9). In this study, CiteSpace is primarily used to analyze the strength, grouping, and time series of keywords.

We used the R package “bibliometrix” to analyze the development trends of popular topics and keywords (10). Compared to some simpler analysis tools, “bibliometrix” can perform more complex data analysis operations, such as modeling and analyzing the heat changes of keywords over time, thereby more accurately predicting the development trends of research hotspots and providing scientific evidence. Additionally, Microsoft Office Excel was used for quantitative analysis of publications.

Journal Impact Factor (IF) and Journal Citation Reports (JCR) data were obtained from Web of Science on April 15, 2024.

## 3 Results

### 3.1 Publication trend

**Figure 2:**
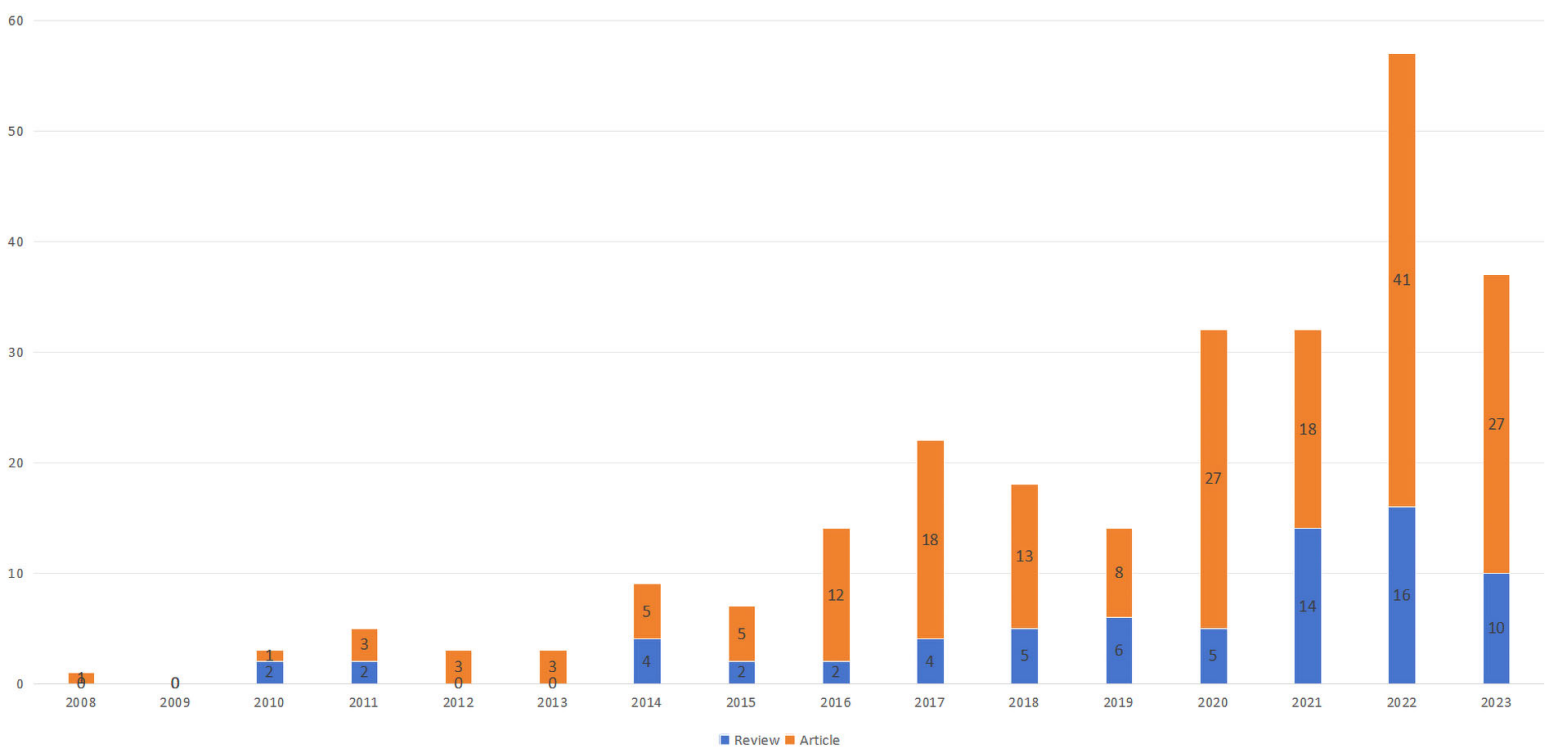
Examination of exosome trends in preeclampsia articles from 2008 to 2023.

As of 2023, there are 257 articles related to exosomes in preeclampsia, including 72 reviews and 185 articles. As shown in **Figure 2**, the first batch of related literature was published in 2008, and the following 6 years (2008 - 2013) saw fewer publications, with an average of 2.5 per year, at the beginning stage of exosome research in preeclampsia. From 2014 to 2023, there was a fluctuating increase in the number of publications, averaging 24.2 per year. Notably, between 2019 and 2022, there was a significant rise in the number of articles published, suggesting that exosomes are gaining prominence in the study of preeclampsia.

### 3.2 Country and institutional distribution analysis

These publications came from 48 countries/regions and 435 institutions. As shown in **Table 1**, among the top 10 countries/regions and institutions engaged in the study of preeclamptic exosomes, China ranked first with 89 publications, followed by the United States (46 publications), England (32 publications), Australia (27 publications), and Chile (16 publications); the top three institutions with the largest number of publications were located in England, China, and the United States, with the University of Oxford (21 publications) being the most published institutions, followed by the University of Queensland (16 publications), the University of Auckland (12 publications), Nanjing Medical University, Tongji University, and the University of Toronto all with 8 publications.

**Table 1:** Top 10 nations and organizations involved in exosome research connected to preeclampsia.

Country/Region	Count	Percent(%)	Institute	Count	Percent(%)
China	89	34.63	University of Oxford	21	8.171
USA	46	17.899	University of Queensland	16	6.226
England	32	12.451	University of Auckland	12	4.669
Australia	27	10.83	University of Concepcion	9	3.502
Chile	16	10.506	University of Toronto	8	3.113
Canada	12	4.669	Nanjing Medical University	8	3.113
New Zealand	12	4.669	Tongji University	8	3.113
Sweden	9	3.502	Ochsner Clinic Foundation	7	2.724
Germany	9	3.502	University of Reading	7	2.724
South Africa	9	3.502	University of KwaZulu-Natal	7	2.724

**Figure 3:**
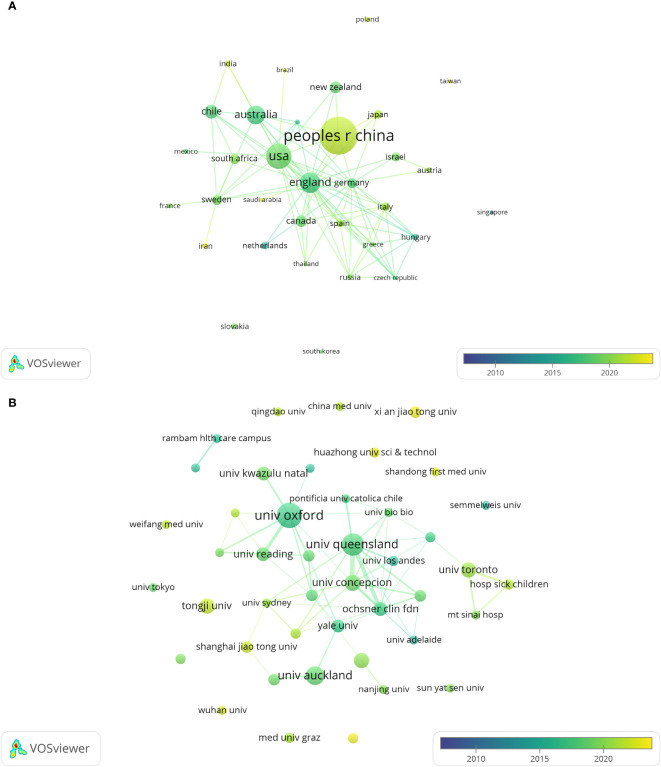
The distribution of countries and institutions publishing research papers on exosomes in preeclampsia. **(A)** Network map of countries/regions publishing exosome research in preeclampsia as shown by VOSviewer. **(B)** Institutional network map of exosome research in preeclampsia shown by VOSviewer.

The national and institutional distribution networks of global articles were illustrated using VOSviewer and normalized via the strength-of-association approach, demonstrating that international collaborations significantly contribute to the research of exosomes in preeclampsia. The national collaboration network (**Figure 3A**) indicates that transnational research collaborations possess notable geographic traits: China constitutes a stable core collaboration circle with the United States, Australia, and New Zealand, while the United States sustains frequent collaborative relationships with the United Kingdom, Chile, and Australia. The average citation frequency of publications resulting from international cooperation is considerably more than that of publications from single-country efforts. The most cited publication in this study was published by Salomon et al. in 2017, representing a collaborative effort among researchers from many countries and research institutes across Europe, North America, and Asia. This multinational collaborative model has markedly expedited advancements in the investigation of exosomes related to the pathogenic mechanisms of preeclampsia by enabling comprehensive integration of research techniques, technical platforms, and theoretical frameworks. At the institutional level (**Figure 3B**), the University of Oxford has formed enduring collaborative partnerships with the University of Reading and the University of KwaZulu-Natal to investigate exosome biomarkers in preeclampsia, whereas the University of Queensland has realized numerous advancements in exosome isolation technology and clinical application through extensive collaborations with the University of Concepcion and the Ochsner Clinical Foundation. Conversely, while Chinese research institutes have established a robust network of domestic collaborations, their transnational collaboration nodes remain relatively sparse, potentially constraining their international academic discourse and technical innovation pathways.

### 3.3 Journal distribution analysis

**Figure 4:**
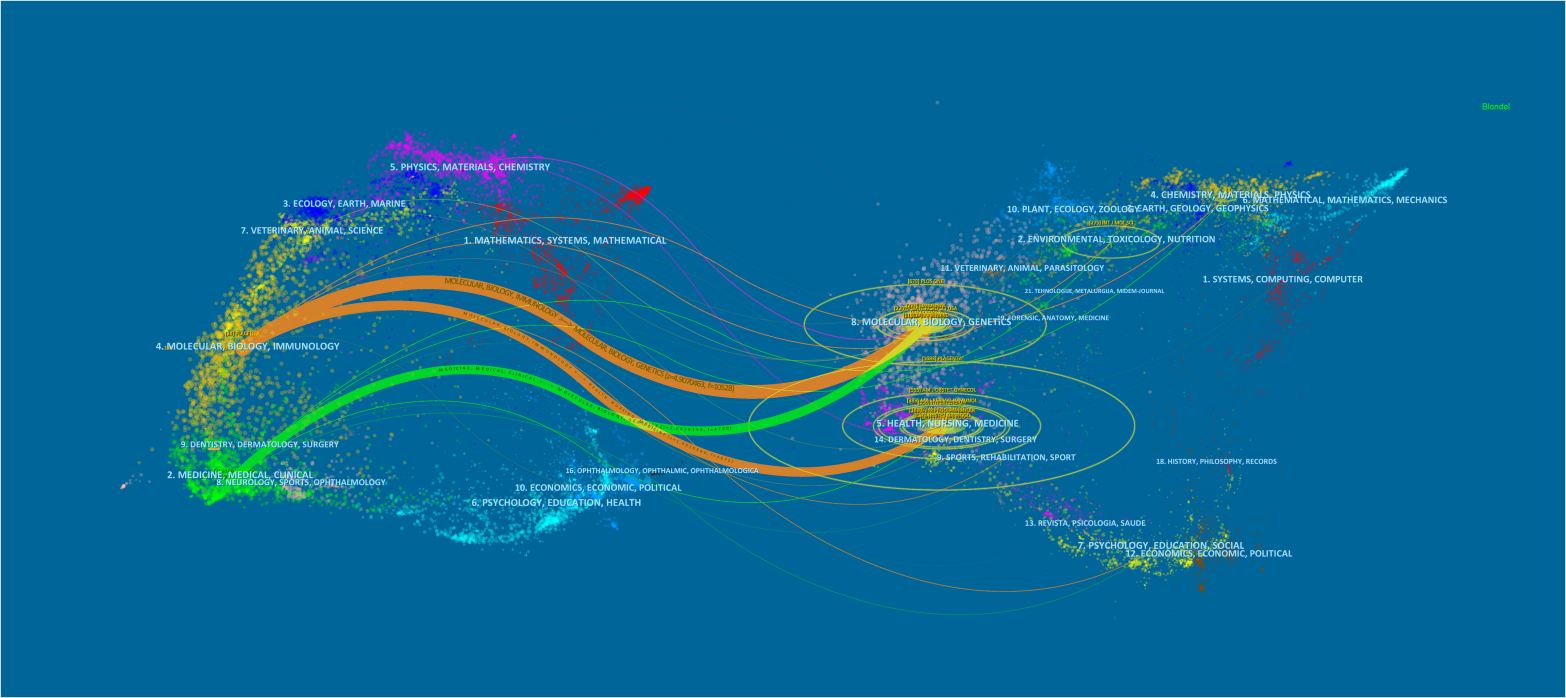
Exosome-related articles in pre-eclampsia from 2008 to 2023 are displayed in a biplot overlay.

Papers on the role of exosomes in preeclampsia were published in 135 journals. As shown in **Table 2**, Placenta published the highest number of research articles with 24, followed by International Journal of Molecular Sciences with 16, and the third highest number of published research articles were Hypertension and Scientific Reports, both with seven. In 2022, the impact factor of these journals ranged from 2.9 to 8.3, Among them, Hypertension had the highest impact factor, while Reproductive Sciences had the lowest. According to the JCR partition analysis, Q1 accounted for 44.44% of this ranking and Q2 accounted for 55.56%. In addition, the top 9 co-cited journals were all cited more than 200 times, with the most cited journals being Placenta (1085citations) and Plos One (680citations), followed by American Journal of Obstetrics and Gynecology (557citations) and American Journal of Reproductive Immunology (383citations). The highest impact factor was found in Journal of Fxtracellular Vesicles (IF= 16, 2022) followed by American Journal of Obstetrics and Gynecology (9.8, 2022). We used VOSviewer to visualize the journal network (**Supplementary Figure 1A**) and co-citation network maps (**Supplementary Figure 1B**), showing active journal citation relationships and co-citation relationships. Additionally, We also used CiteSpace to map the links between cited and co-cited journals (**Figure 4**) and found that the main citation paths were molecular, biology, immunology-molecular, biology, and genetics (z=4.91, f=10528).

**Table 2:** Top 10 journals and co-cited journals studying exosomes in preeclampsia.

Journals	Documents	2022 IF	2022Q	Co-cited Journals	Co-citation	2022 IF	2022Q
Placenta	24	3.8	Q2	Placenta	1085	3.8	Q2
International Journal of Molecular Sciences	16	5.6	Q1	Plos One	680	3.7	Q2
Hypertension	7	8.3	Q1	American Journal of Obstetrics and Gynecology	557	9.8	Q1
Scientific Reports	7	4.6	Q2	American Journal of Reproductive Immunology	383	3.6	Q2
American Journal of Reproductive Immunology	6	3.6	Q2	Hypertension	357	8.3	Q1
Frontiers in Cell and Developmental Biology	6	5.5	Q1	International Journal of Molecular Sciences	275	5.6	Q1
PLoS One	5	3.7	Q2	Journal of Immunology	274	4.4	Q2
Frontiers in Endocrinology	5	5.2	Q1	Scientific Reports	271	4.6	Q2
Reproductive Sciences	5	2.9	Q2	Journal of Extracellular Vesicles	265	16.0	Q1

### 3.4 Author distribution analysis

A total of 1454 authors have been involved in exosomal studies in preeclampsia. The top 9 authors with the most publications and citations are listed in **Table 3**, the most published author is Salomon, Carlos from University of Queensland, Australia (14 publications) followed by Vatish, Manu from University of Oxford, UK (13 publications). The most cited author was Salomon, C from the University of Queensland, Australia (329 citations), followed by Redman, Cwg from the University of Oxford, UK (134 citations). We also screened and mapped the author network (**Appendix Figure 2A**) and co-citation network (**Appendix Figure 2B**) with VOSviewer, and found that there were positive collaborations between authors and co-cited authors.

**Table 3:** Top authors in the field of exosomes and preeclampsia, ranked by number of publications and citations.

Author(publications≥5)	Count	Co-cited author(Citations≥)	Citation
Salomon, Carlos	14	Salomon, C	329
Vatish, Manu	13	Redman, Cwg	134
Rice, Gregory E.	10	Tong, M	109
Tannetta, Dionne	10	Pillay, P	107
Sargent, Ian	7	mincheva-nilsson, l	98
Zhang, Wei	6	Tannetta, D	81
Redman, Christopher	6	Thery, C	74
Chamley, Lawrence W	6	Burton, Gj	72
Chen, Qi	6	Hromadnikova, I	71

### 3.5 Reference distribution analysis

**Table 4** lists the top 10 most frequently cited studies, all with 20 or more citations. Salomon, Carlos et al.’s 2017 publication in the Journal of Clinical Endocrinology & Metabolism, “Placental Exosomes as Early Biomarker of Preeclampsia: Potential Role of Exosomal MicroRNAs Across Gestation” published in the Journal of Clinical Endocrinology & Metabolism in 201711 had the highest number of citations with 58.

**Table 4:** Top 10 documents from citation analysis of publications on exosomes in pre-eclampsia.

Rank	Title	First author	Corresponding author	Source	Publication year	Total citation
1	Placental Exosomes as Early Biomarker of Preeclampsia: Potential Role of Exosomal MicroRNAs Across Gestation	Salomon, Carlos	Salomon, Carlos	Journal of Clinical Endocrinology & Metabolism	2017	62
2	A Gestational Profile of Placental Exosomes in Maternal Plasma and Their Effects on Endothelial Cell Migration	Salomon, Carlos	Salomon, Carlos	Plos One	2014	32
3	Exosomes From Women With Preeclampsia Induced Vascular Dysfunction by Delivering sFlt (Soluble Fms-Like Tyrosine Kinase)-1 and sEng (Soluble Endoglin) to Endothelial Cells	Chang, Xinwen	Wang, Kai	Hypertension	2018	31
4	Minimal information for studies of extracellular vesicles 2018(MISEV2018): a position statement of the International Society for Extracellular Vesicles and update of the MISEV2014 guidelines	Thery, Clotilde	Thery, Clotilde	Journal of Extracellular Vesicles	2018	31
5	Update of syncytiotrophoblast derived extracellular vesicles in normal pregnancy and preeclampsia	Tannetta, Dionne	Tannetta, Dionne	Journal of Reproductive Immunology	2017	29
6	Placental Syncytiotrophoblast-Derived Extracellular Vesicles Carry Active NEP (Neprilysin) and Are Increased in Preeclampsia	Gill, Manjot	Vatish, Manu	Hypertension	2019	28
7	Placental exosomes and pre-eclampsia: Maternal circulating levels in normal pregnancies and, early and late onset pre-eclamptic pregnancies	Pillay, Preenan	Mackraj, Irene	Placenta	2016	28
8	Placenta-derived exosomes: potential biomarkers of preeclampsia	Pillay, Preenan	Mackraj, Irene	International Journal of Nanomeicine	2017	27
9	Placenta-derived exosomes continuously increase in maternal circulation over the first trimester of pregnancy	Sarker, Suchismita	Salomon, Carlos	Journal of Translational Medicine	2014	26
10	Placental exosomes profile in maternal and fetal circulation in intrauterine growth restriction - Liquid biopsies to monitoring fetal growth	Miranda, Jezid	Salomon, Carlos	Placenta	2018	25

**Figure 5:**
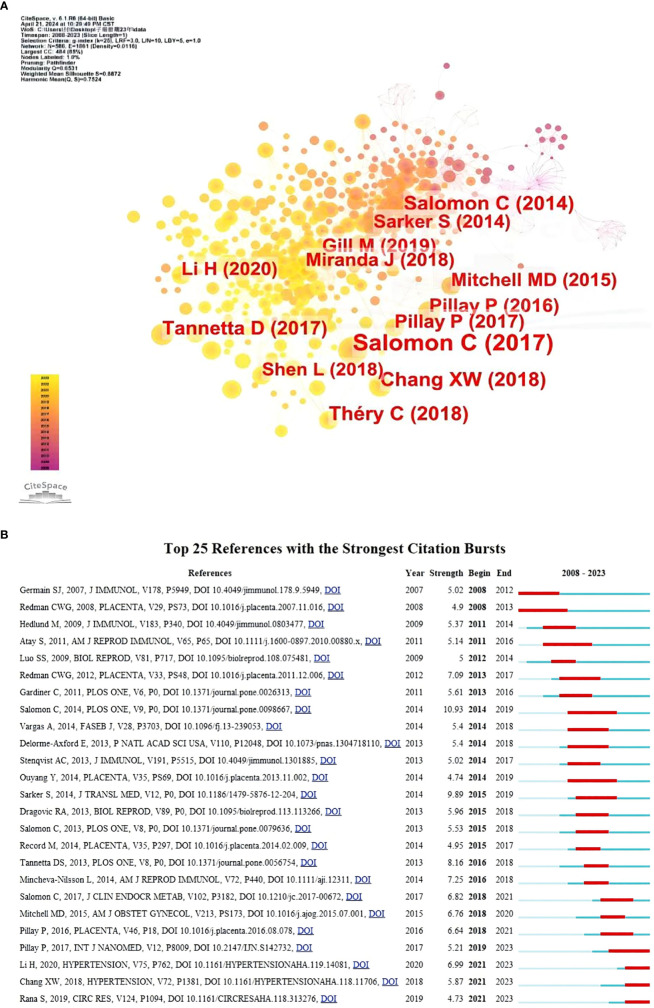
**(A)** Visualization of co-cited references for studies of exosomes in pre-eclampsia. **(B)** Top 25 most cited references. The red bar indicates the high citation rate in the current year.

We used CiteSpace to analyze and visualize the co-citation network of the top 13 shortlisted papers. The key node in the co-citation network is the paper “Placental Exosomes as Early Biomarker of Preeclampsia: Potential Role of Exosomal MicroRNAs Across Gestation” by Salomon, Carlos 2017 in the Journal of Clinical Endocrinology & Metabolism (**Figure 5A**). **Figure 5B** shows the 25 most cited publications based on their citation burst intensity, ranging from 2008 to 2021. The publication “A Gestational Profile of Placental Exosomes in Maternal Plasma and Their Effects on Endothelial Cell Migration” had the highest citation burst intensity of 10.93, and bursts occurred from 2014 to 2019. The article “Placenta-derived exosomes continuously increase in maternal circulation” by Sarker, Suchismita et al. was published in the Journal of Translational Medicine in 2014. The intensity of the paper is 9.89, and it gained citation emergence from 2015 to 2019.

### 3.6 Keyword co-occurrence cluster analysis

**Figure 6:**
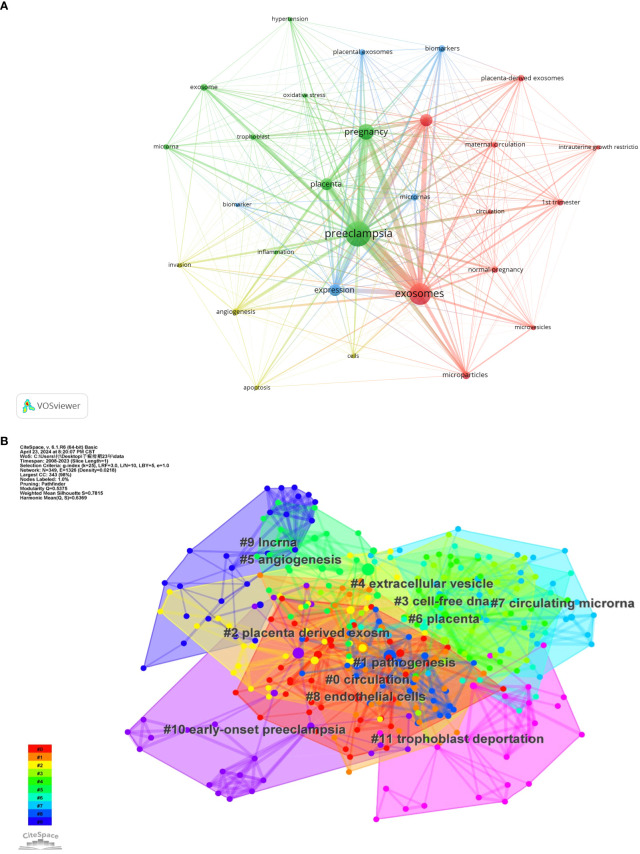
**(A)** Co-occurrence network of keywords by VOSviewer. **(B)** Keyword Cluster Analysis.

Keywords are the main focus of a paper, and analyzing how they appear together helps efficiently identify the trending research topics in a certain sector. **Table 5** displays the top 20 high-frequency keywords and their occurrence rates in the research on exosomes in preeclampsia. The term “extracellular vesicle” was mentioned 76 times, “microparticle” 32 times, and “microRNA” 27 times, making them focal points in the study of exosomes in preeclampsia. The keywords were analyzed using VOSviewer with a threshold set at a minimum of 15 occurrences. A total of 28 keywords were found and categorized into four distinct colored clusters, each indicating various study objectives (**Figure 6A**). The green cluster contained keywords such as preeclampsia, pregnancy, and placentation. The yellow cluster included keywords like angiogenesis and invasion. The blue cluster featured keywords such as expression, microRNAs, and biomarkers. The red cluster included keywords like exosomes, microparticles, and extracellular vesicles.

**Table 5:** Top 25 keywords in documents about exosomes in metabolic disorders.

Rank	Keyword	Count	Rank	Keyword	Count
1	exosm	83	11	maternal circulation	25
2	preeclampsia	78	13	angiogenesis	23
3	extracellular vesicle	76	13	placenta derived exosm	23
4	expression	66	15	gestational diabetes mellitus	22
5	pregnancy	56	16	cell	19
6	microparticle	32	17	endothelial cell	18
7	microrna	27	18	mesenchymal stem cell	16
7	normal pregnancy	27	18	placenta	16
7	1st trimester	27	20	invasion	15
10	biomarker	26	20	circulating microrna	15
11	placental exosm	25	20	circulation	15

By using Citespace’s log-likelihood ratio-based algorithm to cluster the 11 keywords in **Figure 6B** and further refine them, we can classify them into 5 groups: pathogenesis (#0circulation, #1pathogenesis), related diseases (#5angiogenesis, #10early-onset preeclampsia, #11trophoblast deportation), biomarker-related (#2placenta derived exosm, #3cell-free dna, #4extracellular vesicle, #7circulating microrna, #9lncrna), associated cells (#8endothelial cells), and associated organs (#6placenta).

**Figure 7:**
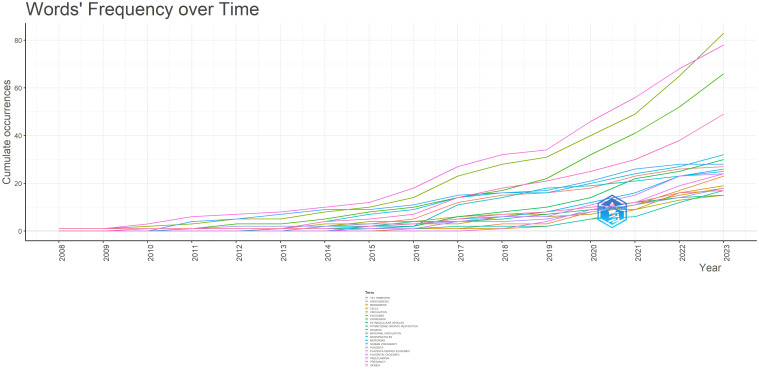
The cumulative growth keywords (top 20).

We conducted a keyword dynamic analysis using R-bibliometrix to analyze hotspot tendencies. **Figure 7** shows the yearly growth rate of the top 20 keywords, indicating that all keywords have experienced an increase since 2009. Preeclampsia, exosomes, and expression have a “J” curve trend, whereas the keywords pregnancy and miRNA are seeing significant growth.

**Figure 8:**
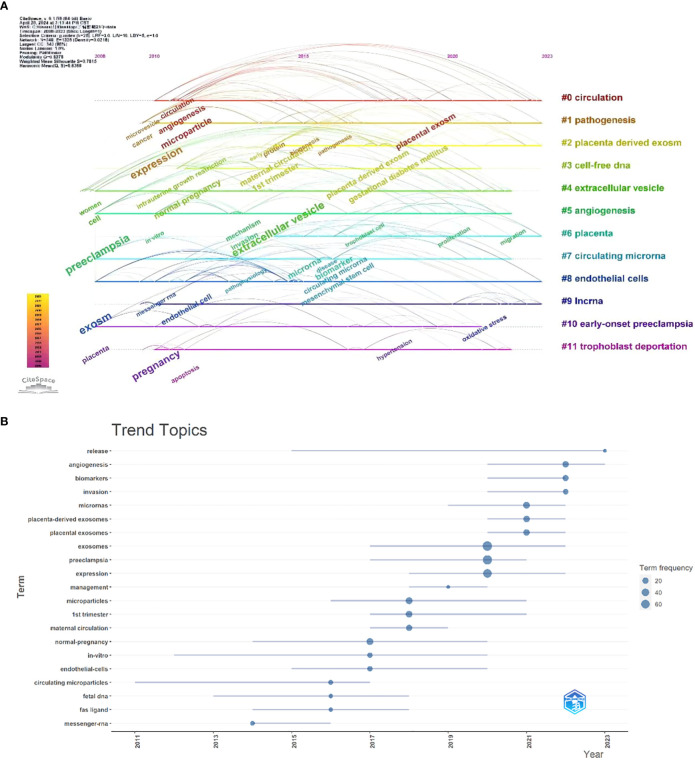
**(A)** Browse graph of CiteSpace timeline visualization associated with exosomes in preeclampsia. Trend topics. **(B)** The X-axis represents the year, while the Y-axis is the cumulate occurrences of the keywords.

We employed Citespace to cluster terms over time, highlighting the focal points and developmental trajectories of exosome research in preeclampsia (**Figure 8A**), and utilized R-bibliometrix for trend theme analysis (**Figure 8B**). Analysis of the two figures reveals that between 2008 and 2010, there was a paucity of research on exosomes in preeclampsia, primarily addressing cellular and preeclampsia-related complications. From 2010 to 2019, the subject garnered significant attention, with investigations concentrating on specific molecular mechanisms linked to exosomes in preeclampsia, emphasizing keywords such as proteins, miRNAs, and angiogenesis. Following 2020, researchers initiated studies on the interplay between placental exosomes and those released by trophoblast cells, utilizing exosomes as biomarkers and therapeutic agents in preeclampsia. Consequently, biomarkers and therapeutic targets emerged as new focal points, and advancements in separation technologies, including ultracentrifugation and size exclusion chromatography, enhanced the purity of exosome isolation. Improvements in exosome detection methodologies, including nanoparticle tracking analysis (NTA) and mass spectrometry, have enhanced the sensitivity and precision of exosome identification (11). Consequently, biomarkers have emerged as a focal point in exosome research pertaining to preeclampsia. The advancement of exosome drug-delivery technology has enhanced drug stability and targeting while minimizing negative effects. In the future, exosomes may serve as a target for personalized therapy, enabling the selection of tailored treatment programs based on individual patient situations. For instance, identifying particular molecules in exosomes enables the provision of tailored pharmacological therapy to patients (5), indicating a shift in research towards practical use.

## 4 Discussion

### 4.1 General information

This study systematically analyzed the research literature on exosomes in the field of preeclampsia up to 2023 based on bibliometric methods. The results showed that since 2008, the annual number of publications in this field has been on a steadily increasing trend. Notably, the growth rate was particularly significant during 2019-2022. China and the United States are the leading countries in this research area, accounting for 52.53% of the global publications. Nanjing Medical University and Tongji University in China, as well as the Oxnard Clinic Foundation in the United States, rank among the top ten research institutions globally.

The relevant research literature is mainly published in academic journals focusing on pregnancy, cell biology, immunology, molecular biology, reproductive biology, and placental research. Placenta is the journal with the highest number of publications, while Hypertension has the highest impact factor in this discipline. Most of the cited journals belong to Q1 and Q2 zones, which fully demonstrates their high influence and excellent quality, laying a solid theoretical foundation for subsequent research work and the exchange and sharing of research findings. At present, research in this field mainly focuses on the basic research level, and more time and effort are needed in the future to apply these findings to clinical practice.

Among the top 10 authors, Carlos Salomon from the University of Queensland in Australia is the most frequently co - cited author in the field of exosomes. He has published a total of 14 papers, making significant contributions to this field. His research focuses on the correlations between exosomes, preeclampsia, gestational diabetes mellitus, hypoxia - induced exosome changes, extracellular vesicles, and angiogenesis during pregnancy (12–17). He emphasized the biological relevance of exosomes and their potential role in pregnancy. Changes in exosome content and concentration may serve as diagnostic markers for identifying pregnant women at high risk of preeclampsia (12).

### 4.2 Knowledge base

Co-cited literature refers to the materials cited by numerous scientific research articles. It usually contains the boundary definition of the research field, the core content of the knowledge system, the evolution of academic viewpoints, and emerging research trends (18). They are regarded as the key or summary literature of this discipline. The knowledge base is a compilation of literature focusing on a specific field or topic. It organizes and presents relevant knowledge by utilizing the co-citation relationship between literatures and provides basic information about the research field. By analyzing the knowledge base, researchers can gain insights into the research dynamics, current trends, and innovative directions of a specific topic, which can provide valuable references and suggestions for their own research work (19). This study demonstrated the scientific basis of exosomes in preeclampsia by identifying the top 10 co - cited references (**Table 4**).

Among them, a paper published by Carlos Salomon in the Journal of Clinical Endocrinology & Metabolism in 2017 is the most frequently cited literature on the topic of exosomes in preeclampsia (12). This study used nanoparticle tracking analysis technology to analyze and identify exosomes in the maternal circulation and successfully created a microRNA library. It was finally found that the concentration and composition of exosomes are expected to be important indicators for diagnosing preeclampsia. Meanwhile, the paper he published in Plos One is the second most cited literature (20). This study explored the release characteristics and biological activities of exosomes derived from placental cells in maternal plasma at different stages of fetal development. The results showed that changes in exosome characteristics may be clinically helpful for diagnosing placental dysfunction.

The third co-cited study was written by Chang, Xinwen, et al. and published in Hypertension in 2018 (21). The results showed that exosomes from preeclamptic patients can directly induce vascular dysfunction in a mouse model, leading to adverse preeclampsia outcomes. Exosomes are believed to help transfer soluble vascular endothelial growth factor receptor-1 (sFlt-1) and soluble endoglin (sEng) to endothelial cells, thus causing vascular dysfunction and various problems in preeclamptic patients.

The fourth co-cited literature outlined the key information required for studying extracellular vesicles (EVs), including the isolation, concentration, quantification, proteomic analysis, single-vesicle analysis, functional research of extracellular vesicles, and the unique roles of various types of extracellular vesicles (22). The fifth co-cited paper used nanoparticle tracking analysis, flow cytometry, and mass spectrometry to study the role of syncytiotrophoblast-derived extracellular vesicles (STBEVs) in normal pregnancy and preeclampsia. The possibility of STBEVs as placental pathology indicators and therapeutic targets for preeclampsia was explored (23). The sixth co-cited literature studied the up-regulation of neutral endopeptidase (NEP) in the placenta and peripheral circulation of preeclamptic patients and the enhanced activity of related STBEVs in these patients. The research results showed that NEP may be a potential therapeutic target for preeclampsia and may also play a role in the diagnosis of preeclampsia (24).

The seventh co - cited literature is a study published in Placenta in 2016 by Preenan Pillay, et al. This study explored the impact of placental exosomes on pregnancy-induced hypertension (preeclampsia). The research results showed that the total number of exosomes in preeclamptic patients was higher, but the proportion of placental-derived exosomes was lower. In addition, there were differences in the levels of placental-derived exosomes between preeclamptic and late-onset preeclamptic patients. These results provided the first information that placental-derived exosomes may be involved in preeclampsia and suggested that exosomes could be used as diagnostic indicators for this disease (25). The eighth co-cited review discussed the potential of placental-derived exosomes as biomarkers for the diagnosis and prediction of preeclampsia outcomes, as well as the possibility of being potential therapeutic strategies for this disease (26). The ninth co-cited paper discussed the finding that the number of placental-derived exosomes in maternal blood increased steadily during the first three months of normal pregnancy. This finding provides the possibility for early detection of pregnancy complications and the development of new clinical intervention methods (27). The tenth co-cited literature demonstrated the correlation between the concentration of placental-derived exosomes in the maternal and fetal circulation and fetal development. This relationship indicates that these exosomes can be used as biomarkers to evaluate placental function and monitor fetal growth restriction (28).

The top 10 co-cited papers provided valuable insights into the characteristics, composition, biological functions, and targeted delivery of exosomes. They laid a scientific foundation for the application of exosomes in disease diagnosis, treatment monitoring, and the development of potential therapeutic methods, establishing a solid foundation for this field.

### 4.3 Hotspots and frontiers

Through the methods of keyword clustering and topic analysis, the hotspots and frontiers of current research on exosomes in preeclampsia were deeply analyzed and summarized.

#### 4.3.1 Exosome sources

Placental exosomes are currently the most widely studied objects. Trophoblast cells, as their main source, play a crucial role in the research of immune regulation and pregnancy -related issues. Placental exosomes can induce immunosuppressive reactions by transferring exosomal proteins to T cells. At the same time, placental-derived miRNAs play a vital role in the molecular signal transduction process throughout pregnancy. For example, by down - regulating the expression of CD44, they can effectively reduce the invasiveness of cells. In recent years, umbilical cord-derived exosomes have gradually become the focus of research. It has been found that miRNAs in umbilical cord blood exosomes, especially has-miR-125a-5p, are closely related to preeclampsia. Its abnormal expression may disrupt the normal formation of placental blood vessels by affecting the growth and movement of placental cells. In addition, patient plasma exosomes also contain specific miRNAs, which are closely related to stress responses and cell-junction regulation. In summary, exosomes from different mother-cell sources profoundly affect the occurrence and development of preeclampsia through a variety of complex mechanisms.

In the research of preeclampsia, placental exosomes have been the most widely studied. Trophoblast cells, as their main source, play a key role in the study of immune regulation and pregnancy issues. Placental exosomes *in vivo* have immunosuppressive properties. They can induce immunosuppression by reducing the production of Th1-type cytokines in activated peripheral blood mononuclear cells (PBMCs) and by transferring exosomal proteins to T cells (29). Placental miRNAs in exosomes are crucial in the molecular signal transduction throughout pregnancy. For example, they may reduce the invasive ability of trophoblast cells by down-regulating the expression of CD44 (30). Placental exosomes containing miR-29b may trigger trophoblast cell death and impede their invasive and angiogenic capabilities (31). Trophoblast cells in the placenta promote communication between the mother and the fetus by releasing exosomes and particles. This process forms a complex information-sharing network that plays a role in immune regulation and promoting the healthy growth of the fetus throughout pregnancy (32). In recent years, umbilical cord exosomes have become the focus of preeclampsia research. There is an important connection between has-miR-125a-5p in umbilical cord blood exosomes and preeclampsia. The abnormal expression of this miRNA in pregnant women with preeclampsia can affect the growth and movement of placental cells, leading to obstruction of placental angiogenesis (33).

Exosomes in the placenta transport bioactive chemicals that can regulate the growth, death, and activity of placental cells, affect the formation and function of the placenta, and thus affect the health of the mother and the fetus (34). Exosomes released by human umbilical cord mesenchymal stem cells (HUCMSCs) containing miR-101 can be transported to placental trophoblast cells. Studies have shown that miR-101 plays a role in the formation of preeclampsia by reducing the expression of BRD4 protein, resulting in the inhibition of the NF-κB/CXCL11 signaling pathway and ultimately enhancing the proliferation and movement capabilities of placental trophoblast cells (35). Mesenchymal stem cell - derived exosomes enhance the movement of vascular endothelial cells and the formation of new blood vessels by transporting molecules such as miR-302a and MMP2. This process helps to alleviate the adverse outcomes of the preeclampsia mouse model, such as reducing blood pressure and increasing fetal birth weight (36).

Plasma exosomes of preeclamptic patients contain specific miRNAs, which can serve as signaling molecules related to stress responses and cell-junction regulation (37, 38). Exosomes may produce cytokines and inflammatory mediators, affecting the immune-inflammatory response and promoting the occurrence of preeclampsia. The bioactive chemicals transported by exosomes can directly affect vascular endothelial cells, causing vasoconstriction and functional problems, ultimately leading to hypertension and vascular damage (34).

In summary, exosomes from different mother-cell types play roles in the occurrence and development of preeclampsia through various pathways.

#### 4.3.2 Exosome isolation and characterization techniques

Exosomes, as critical mediators of intercellular communication, require precise isolation and characterization to deeply investigate their functions and roles in diseases (39). Reliable techniques for exosome isolation and characterization enable researchers to obtain high-purity exosome samples, providing robust support for subsequent functional studies.

Exosome isolation techniques are diverse, with commonly used methods including differential centrifugation, density gradient centrifugation, precipitation, filtration, and size-exclusion chromatography (**Table 6**) (40, 41). Each method has its advantages and limitations, and selecting the appropriate technique based on experimental objectives and sample types can lead to more ideal results. For example, differential centrifugation is simple to perform but may introduce impurities, while density gradient centrifugation yields higher-purity exosomes but is more complex and time-consuming.

**Table 6:** Exosome isolation and characterization techniques.

Technique	Description	Advantages	Disadvantages
Differential Centrifugation	Stepwise removal of impurities by centrifugation at different speeds to isolate exosomes	Simple operation, widely used	May introduce other particles, lower purity
Density Gradient Centrifugation	Separation based on density differences between exosomes and other components in a gradient medium	High purity of exosomes	Complex operation, time-consuming
Precipitation	Use of polymers to precipitate exosomes	Simple operation, suitable for large sample processing	May co-precipitate impurities, affecting purity
Filtration	Separation of exosomes using specific pore-size filters	Can select pore size based on needs, simple operation	May clog filters, sample loss
Size-Exclusion Chromatography	Separation based on size differences of exosomes	Provides relatively uniform samples, reduces impurity contamination	Long separation time, may lose some exosomes
Immunoaffinity Capture	Use of antibodies to specifically bind exosome surface markers	High specificity, suitable for enriching specific exosome phenotypes	High cost, complex operation, antibodies may affect exosome structure and function
Nanoparticle Tracking Analysis (NTA)	Measurement of exosome size and concentration by analyzing Brownian motion of nanoparticles	Fast, intuitive, provides size distribution and concentration information	Difficult to distinguish particles of similar size
Transmission Electron Microscopy (TEM)	Observation of exosome morphology and structure using electron microscopy	Intuitive visualization of exosome morphology, provides high-resolution images	Complex sample preparation, limited observation area
Western Blot	Detection of exosome protein markers such as CD63, CD9, CD81	Qualitative or semi-quantitative analysis of exosome protein markers	Complex operation, limited sensitivity
Enzyme-Linked Immunosorbent Assay (ELISA)	Quantitative detection of exosomes using antibodies specific to exosome surface markers	High sensitivity, quantitative detection of target proteins	Possible antibody cross-reactivity, affecting result accuracy

Exosome characterization can be determined through various techniques, such as nanoparticle tracking analysis (NTA), transmission electron microscopy (TEM), Western blot, and enzyme-linked immunosorbent assay (ELISA) (**Table 6**) (41). These methods provide comprehensive analysis of exosome morphology, size, concentration, and protein markers from different perspectives, further confirming their origin and purity.

By integrating multiple isolation and characterization techniques, researchers can accurately understand the biological properties of exosomes and their roles in diseases, laying the foundation for the development of exosome-based diagnostic and therapeutic strategies.

#### 4.3.3 Cargo in exosomes

Exosomes are nanoscale membrane vesicles with a diameter typically ranging from 30 to 150 nm (42). They carry a variety of bioactive molecules, including miRNAs, proteins, and lipids. These cargoes play a crucial role in intercellular communication and can regulate the functions of recipient cells. In the context of preeclampsia (PE), the specific cargoes carried by exosomes affect processes such as placental development, angiogenesis, and immune regulation through multiple pathways, thus contributing to the occurrence and development of the disease (**Table 7**).

**Table 7:** Functional roles of cargo carried by exosomes in the pathogenesis of preeclampsia.

Cargo Type	Specific Molecule	Functional Role	Abnormal Manifestations in Preeclampsia	References
miRNA	miRNA-155	It interacts with the 3’untranslated region of eNOS mRNA, inhibits the production of eNOS mRNA, reduces the stability of eNOS mRNA, and may lead to impaired endothelial cell function	The expression level is significantly increased.	(44)
	miRNA-222	It reduced the production of nitric oxide, which is consistent with the pathogenic process of preeclampsia	The expression is significantly decreased.	(44)
	miRNA-199a-5p	It acted on the SIRT1 gene, impairs endothelial cell function	It is significantly increased in plasma exosomes.	(45)
	miRNA-486-5p	It reduced the expression of the ARHGAP5 gene and hindering the migration and invasion of placental trophoblast cells	Its expression in the blood of pregnant women is increased.	(46) (47)
	miRNA-18b-3p	Exosomes derived from human umbilical cord mesenchymal stem cells reduce the systolic blood pressure and 24-hour urinary protein levels of preeclamptic rats by expressing it, increase the placental weight, reduce the levels of inflammatory factors (such as TNF-α, IL-1β, and IL-6) in the serum and placental tissue, and inhibit cell apoptosis, ultimately suppressing the progression of preeclampsia	/	(48)
	miRNA-520c-3p	In the early stage of pregnancy, it in embryonic exosomes interacts with CD44 and hyaluronic acid, which may regulate the communication between plasma cell exosomes	/	(49)
	miRNA-126	Regulates angiogenesis, inhibits Spred-1 expression, promotes endothelial cell proliferation and migration	Downregulated in preeclampsia patients, leading to abnormal placental angiogenesis and placental ischemia	(15)
	miRNA-210	Regulates cellular adaptation to hypoxia, participates in angiogenesis and inflammatory responses	Upregulated under hypoxic conditions, potentially involved in the pathogenesis of preeclampsia	(31)
Protein	sFlt-1	Inhibits angiogenesis, binds to PlGF and VEGF, causing vascular endothelial dysfunction	Upregulated in preeclampsia patients, inhibiting angiogenesis and leading to placental ischemia	(43)
	sEng	Inhibits angiogenesis, interferes with TGF-β signaling, causing vascular endothelial dysfunction	Upregulated in preeclampsia patients, inhibiting angiogenesis and leading to placental ischemia	(43)
	PLAP	Specific marker protein for placental-derived exosomes, reflects placental function	Significantly elevated in preeclampsia patients, indicating impaired placental function	(50)
	BRD4	miR-101 plays a role in the formation of preeclampsia by reducing its expression, resulting in the inhibition of the NF-κB/CXCL11 signaling pathway and ultimately enhancing the proliferation and movement capabilities of placental trophoblast cells	/	(35)
	FOXO1, let-7b, AKT-related proteins	Exosomes released by mesenchymal stem cells transport H19 to trophoblast cells, resulting in decreased let-7b expression, increased FOXO1 expression, and activation of the AKT signaling pathway. This process enhances the invasive and migratory capabilities of trophoblast cells and inhibits their apoptosis	/	(51)
Lipid	Sphingomyelin	Participates in cell signaling and membrane fluidity regulation, affects apoptosis and inflammatory responses	Abnormal levels in preeclampsia patients, potentially exacerbating apoptosis and inflammatory responses	(52)
	Ceramide	Participates in cell signaling and membrane stability regulation, affects apoptosis and inflammatory responses	Abnormal levels in preeclampsia patients, potentially exacerbating apoptosis and inflammatory responses	(53)

miRNAs are a class of non-coding RNAs that regulate gene expression by binding to target mRNAs, inhibiting their translation or promoting their degradation. In preeclampsia, the abnormal expression of miRNAs carried by exosomes affects placental development and angiogenesis. For example, the expression of miR-126 is downregulated in preeclamptic patients, leading to abnormal placental angiogenesis and subsequent placental ischemia and hypoxia. In addition, the expression of miR-210 increases under hypoxic conditions and may be involved in the pathogenesis of preeclampsia (31).

Proteins in exosomes are involved in various biological functions, such as cell adhesion, signal transduction, and immune regulation. In preeclampsia, the proteins carried by placental-derived exosomes, such as sFlt-1 and sEng, are significantly upregulated, inhibiting angiogenesis and leading to vascular endothelial dysfunction (43). Moreover, placental alkaline phosphatase (PLAP), a specific marker protein of placental - derived exosomes, has a significantly increased concentration in preeclamptic patients, reflecting impaired placental function. Lipids in exosomes are involved in intercellular signal transduction and the regulation of membrane fluidity. In preeclampsia, there is a disorder of lipid metabolism, and the levels of lipids in exosomes, such as sphingomyelin and, are abnormal. This may affect cell apoptosis and inflammatory responses, further exacerbating the condition of preeclampsia.

These cargoes carried by exosomes, through a complex interaction network, jointly affect the development and function of the placenta, leading to pathological changes such as vascular endothelial dysfunction and inflammatory responses.

#### 4.3.4 Target cells and effects

Ma, Ruixia, et al. found that miR-486-5p in exosomes released by human pulmonary vein endothelial cells (HPVECs) inhibits the proliferation, migration, and invasion of trophoblast cells by targeting IGF1. Overexpression of IGF1 can effectively counteract the effect of the miR-486-5p inhibitor in HPVECs exosomes on the function of trophoblast cells (47). Exosomes released by mesenchymal stem cells (MSCs) mainly transport H19 into trophoblast cells, resulting in decreased let-7b expression, increased FOXO1 expression, and activation of the protein kinase B (AKT) signaling pathway. This process enhances the invasive and migratory capabilities of trophoblast cells and inhibits their apoptosis (51). Studies have shown that extracellular vesicles (PE-EVs) from preeclamptic patients with pulmonary embolism can increase the expression of gene markers related to M1-type macrophages and decrease the expression of the M2-type macrophage marker CD163, indicating that PE-EVs may affect the development of pulmonary embolism by regulating macrophage polarization. In animal experiments, PE-EVs induced preeclampsia-like symptoms in pregnant mice, and these symptoms were alleviated after macrophage reduction, further demonstrating the important role of macrophages in the development of preeclampsia (54).

Exosomes carrying cargo released by mother cells have an impact on the target cells of preeclampsia and play a significant role in the development of this disease.

#### 4.3.5 Biomarkers

In recent years, the progress of separation and purification technologies (as shown in the table) has made it possible to extract exosomes from various body fluids such as serum, plasma, urine, milk, and saliva, opening a new era of disease detection. A variety of miRNAs have been considered as potential biomarkers for diagnosing and predicting the outcomes of preeclampsia.

Anat Aharonmi found in the experiment that the levels of RNA-16 and miRNA-210 in preeclamptic patients were moderately negatively correlated with maternal systolic blood pressure, and the expression of miRNA-29b in placental tissues and primary placental cells of preeclamptic patients was significantly increased (31). Another study found that the expression of miR-150-3p was negatively correlated with CHPF in the placental vascular tissue of preeclamptic patients (55). Teresa Maria Seccia, et al. reported that the expression levels of miR-210 and miR-155 were significantly increased in preeclamptic patients. miR -210 directly acts on signal transducer and activator of transcription 6 (STAT6) to reduce the production of IL-4, while miR-155 is related to the regulation of renin-transcription activator (RET) and is related to the regulation of the expression of renin-angiotensin system factors (56). Rosana Navajas, et al. found that the exosomal expression of specific proteins such as SVEP1, VCAN, RELN, and PZP showed similar quantitative changes in samples collected at different time points (57). The detection of specific exosomes in blood, urine, or other physiological body fluids provides new possibilities for early detection (5).

Eric Devor, et al. found that in preeclamptic patients during pregnancy, the expression of specific miRNAs showed unique changes at different stages of pregnancy. For example, in the early stage of pregnancy, the expression of miR-134, miR-196b, miR-376c, miR- 486-3p, and miR-590-5p in preeclamptic patients was higher than that in normal pregnant women, but lower in the subsequent pregnancy. In addition, miR-302c, miR-346, and miR -618 showed the opposite expression pattern in preeclamptic patients (58).

In summary, exosomes and their contents extracted from body fluids are expected to be reliable biomarkers for preeclampsia.

#### 4.3.6 Biomarkers

Recent progress in isolation and purification techniques have enabled the extraction of exosomes from various bodily fluids such as serum, plasma, urine, milk, and saliva. This has ushered in a new age in disease detection. Several miRNAs have been recognized as possible biomarkers for diagnosing and predicting the outcome of preeclampsia.

Anat Aharonmi and colleagues discovered in their experiment that the levels of RNA-16 and miRNA-210 in individuals with pre-eclampsia showed a moderate negative correlation with maternal systolic blood pressure. Additionally, they observed a significant increase in miRNA-29b expression in placental tissues and primary placental cells of patients with pre-eclampsia (31).In placental vascular tissues from individuals with preeclampsia, another study discovered a negative correlation between miR-150-3p expression and CHPF (55). Teresa Maria Seccia et al. reported that miR-210 and miR-155 their expression levels were significantly elevated in patients with preeclampsia. miR-210 directly targets signal transducer and activator of transcription factor 6 (STAT6), thereby reducing IL-4 production, while miR-155 is associated with the regulation of renin-activator of transcription (RET), and miR-155 is associated with the regulation of RET. 155 is associated with the regulation of the expression of factors of the renin-angiotensin system (56).By analyzing the configuration of plasma exosomal miRNAs, it was found that pregnant women with sPE showed significant changes in plasma exosomal miRNA expression compared to normal pregnant women: the expression of 15 miRNAs was up-regulated, and 14 were down-regulated.36 Navajas, Rosana et al. found that the exosomal expression of specific proteins such as SVEP1, VCAN, RELN, and PZP showed similar quantitative changes in samples collected at different time points (57).Particular exosomes may be identified in blood, urine, or other physiological fluids, offering new possibilities for early detection (5).

Eric Devor et al. found that in patients with gestational pre-eclampsia, the expression of specific miRNAs showed unique variations across pregnancy stages. For example, miR-134, miR-196b, miR-376c, miR-486-3p, and miR-590-5p showed higher expression in patients with pre-eclampsia than in women with normal pregnancies during the first trimester of gestation, but showed lower expression in subsequent trimesters. In addition, miR-302c, miR-346, and miR-618 showed the opposite expression pattern in patients with pre-eclampsia (58).

In conclusion, exosomes extracted from bodily fluids and their contents are anticipated to serve as reliable biomarkers for preeclampsia.

#### 4.3.7 Treatment

Exosomes exhibit significant potential value in the clinical application of preeclampsia. They offer new strategies for early disease diagnosis, precise prognosis assessment, and targeted treatment through various mechanisms.

Exosomes carry a variety of biomarkers, providing a new approach for early diagnosis of the disease. Some studies have shown that the concentrations of total exosomes and placenta - derived exosomes in the plasma of pregnant women with preeclampsia are significantly higher than those in normal pregnant women. Detecting the concentration of exosomes in the plasma of pregnant women during the first trimester (11-14 weeks) is of great significance for identifying asymptomatic women who will develop preeclampsia later. In addition, miRNAs carried by exosomes, such as has-miR-486-1-5p and hsa - miR -486-2-5p, are significantly upregulated in the exosomes of pregnant women with preeclampsia. Combining with the detection of exosome concentration can significantly improve the accuracy of early diagnosis.

The biological information carried by exosomes can reflect the disease progression and severity of preeclampsia. When the placental function is impaired, more exosomes are released into the maternal circulation. The changes of certain proteins and miRNAs in them are closely related to the disease progression. The overexpression and secretion of sFlt-1 in exosomes inhibit the proliferation, migration, and lumen formation of human umbilical vein endothelial cells (HUVECs), further inducing vascular dysfunction in preeclampsia. Monitoring the changes in its level can effectively evaluate the disease severity and predict the risk of maternal-fetal complications. Using specific inhibitors or antibodies to block the relevant pathways can inhibit the up-regulation of sFlt-1 expression, thereby treating preeclampsia (43).

Exosomes have advantages such as low immunogenicity, good biocompatibility, and targeting ability, providing a new way for targeted therapy. Exosomes can transport functional biomolecules and serve as carriers for drug delivery. By binding to specific receptors, they can be delivered directionally to target cells, thus achieving precise transmission of information and substances (59). Long non-coding RNAs (such as ZEB2-AS1) in exosomes released by placental tissue play a role in maintaining a healthy pregnancy. They promote immune tolerance at the maternal - fetal interface by controlling the movement and function of immune cells (60). Mesenchymal stem cells (MSCs) have anti-inflammatory, immunomodulatory, tissue-repairing, and regenerative effects and can be used to treat various diseases. In *in-vitro* experiments, exosomes derived from MSCs promote the migration, invasion, and proliferation of trophoblast cells and reduce cell apoptosis by delivering miR-139-5p to trophoblast cells and regulating the ERK/MMP-2 pathway (23). Stem-cell-derived exosome therapy may have potential benefits in treating preeclampsia by changing the characteristics of specific cells and improving the unhealthy placenta. MSC-derived exosomes can be genetically modified to endow them with unique targeting properties, thus better targeting the treatment of preeclampsia (27). miRNA-548c -5p prevents preeclampsia by targeting the PTPRO gene and blocking the NF-κB signaling pathway to reduce the inflammatory response. Experiments such as Western blotting have found that exosomes derived from human umbilical cord mesenchymal stem cells (hucMSC-Ex) improve the pathological changes of the placenta in preeclamptic rats by reducing placental thickness, edema, inflammatory cell infiltration, and giant cell hyperplasia (61).

## 5 Limitations

We retrieved literature on exosomes in preeclampsia from the WoSCC database and conducted a simultaneous analysis using three bibliometric techniques. However, our research has limitations. Software constraints prevented us from doing correlation analysis, such as co-citation analyses, on databases like PubMed owing to a lack of reference information. Consequently, we collected data from a single database. This research may have been affected by bias, resulting in less thorough and reliable conclusions. Furthermore, VOSviewer, CiteSpace, and bibliometrix are not sufficient substitutes for systematic searches. Therefore, precise literature analysis should rely on software analyses that integrate particular literature to create a knowledge graph. However, visualization-based literature analysis remains a valuable tool for researchers to comprehend the key areas of focus and possible issues related to exosomes in preeclampsia.

## 6 Conclusion

Exosomes are gaining significance in research and prospective applications related to preeclampsia. This field of study is rapidly expanding, with robust international cooperation, particularly between China and the United States. Salomon, Carlos has made substantial contributions to several important articles and is the most often mentioned author in this research. Current studies mostly concentrate on fundamental cellular and miRNA investigations. It is crucial to shift emphasis towards applying research findings to clinical settings for diagnosing and treating preeclampsia using exosomes. This work offers the first systematic bibliometric analysis of articles concerning exosomes in preeclampsia, presenting an unbiased and thorough summary for researchers in the area, and a useful point of reference for other researchers.
