# Supplementary figures and images for: Knowledge mapping of exosomes in preeclampsia: a bibliometric analysis (2008-2023)
Source: Front Endocrinol (Lausanne). 2025 Mar 4;16:1546554. doi: 10.3389/fendo.2025.1546554 (PMC11913699; doi:10.3389/fendo.2025.1546554)

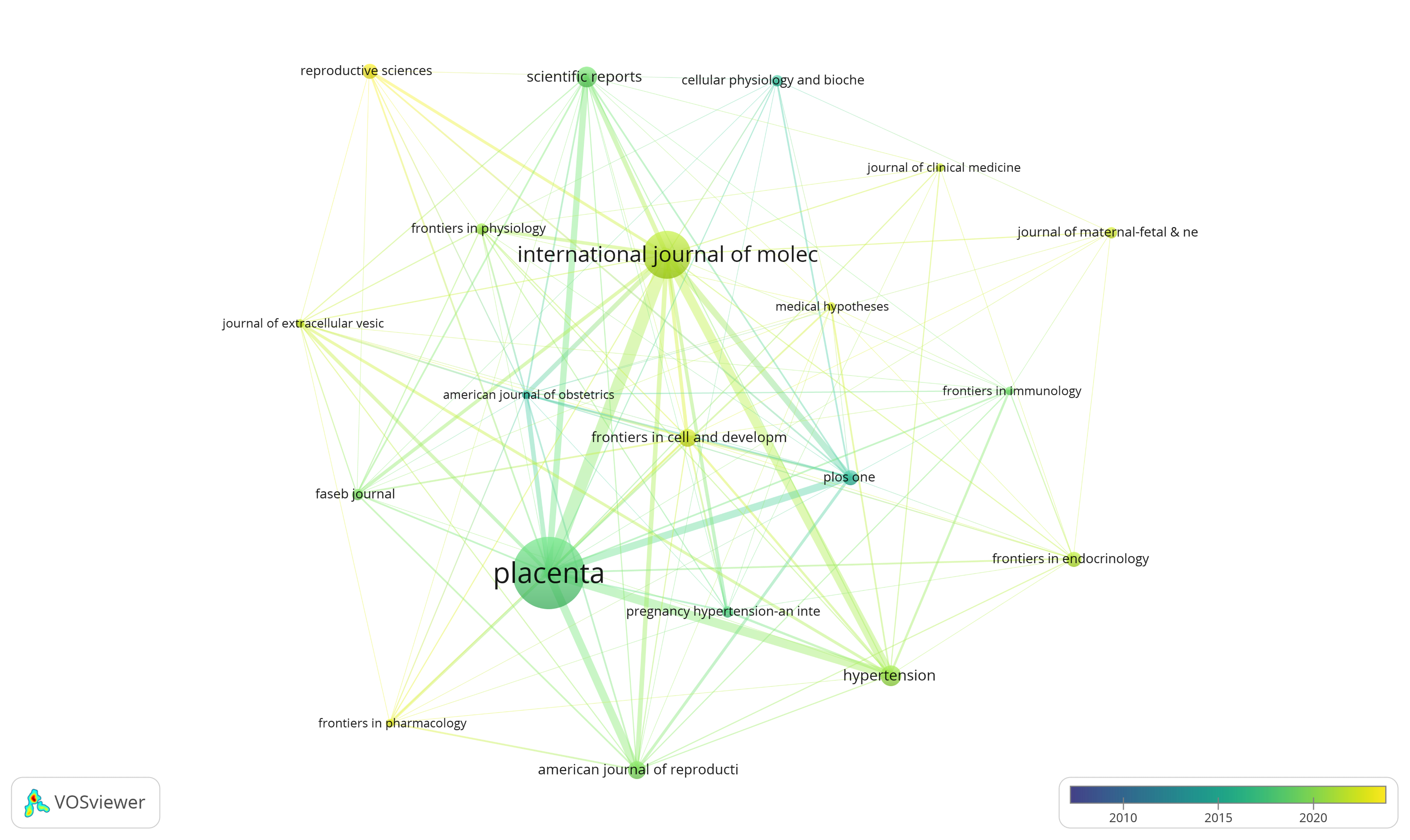

Supplement: Supplementary file 1 [file DataSheet1.zip › Image 1A.tif]

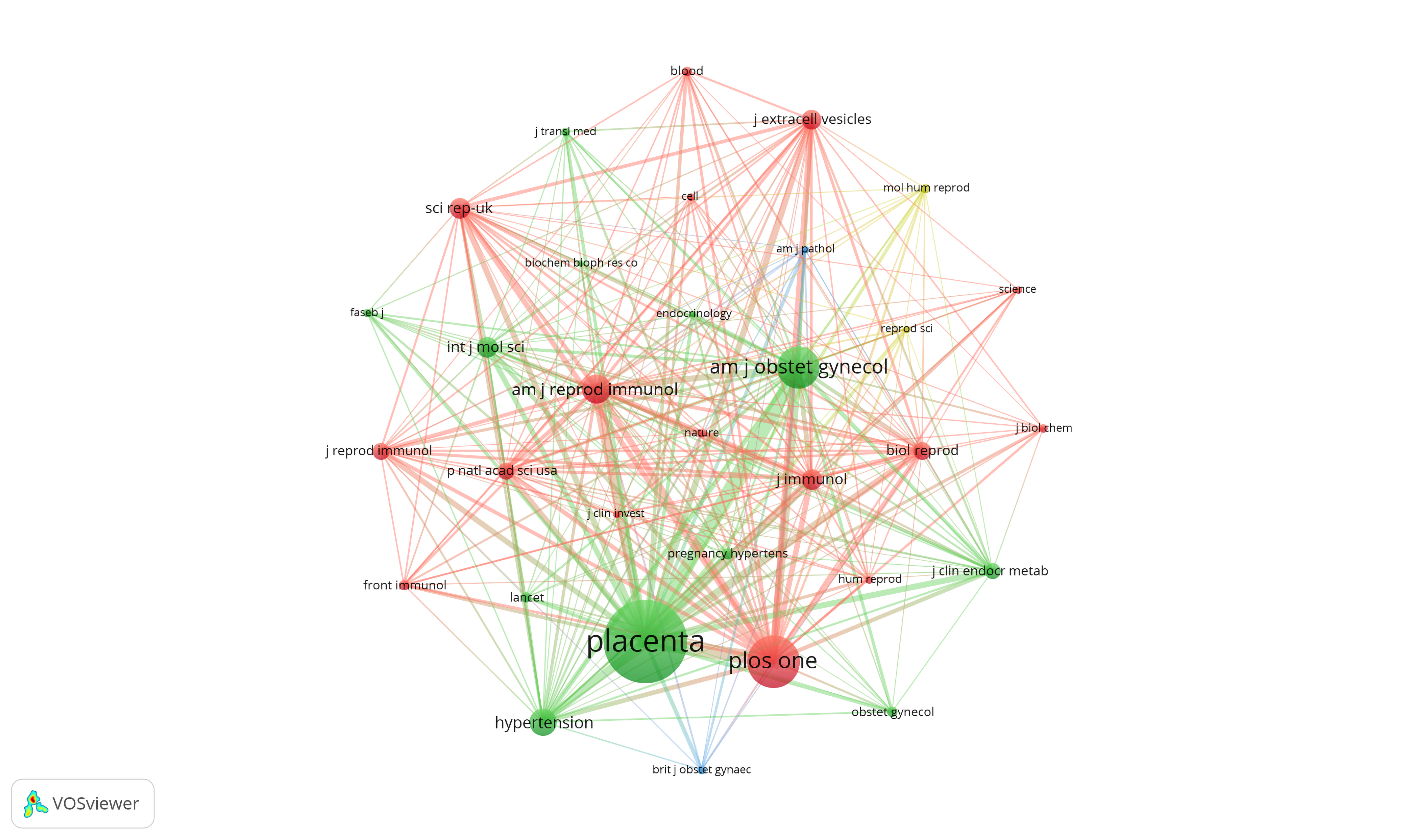

Supplement: Supplementary file 1 [file DataSheet1.zip › Image 1B.tif]
